# Differential physio-biochemical and yield responses of *Camelina sativa* L. under varying irrigation water regimes in semi-arid climatic conditions

**DOI:** 10.1371/journal.pone.0242441

**Published:** 2020-12-02

**Authors:** Zeeshan Ahmed, Junhe Liu, Ejaz Ahmad Waraich, Yan Yan, Zhiming Qi, Dongwei Gui, Fanjiang Zeng, Akash Tariq, Muhammad Shareef, Hassan Iqbal, Ghulam Murtaza, Zhihao Zhang

**Affiliations:** 1 Xinjiang Institute of Ecology and Geography, Chinese Academy of Sciences, Xinjiang, China; 2 Cele National Station of Observation and Research for Desert-Grassland Ecosystem, Xinjiang Institute of Ecology and Geography, Chinese Academy of Sciences, Xinjiang, China; 3 Xinjiang Key Laboratory of Desert Plant Root Ecology and Vegetation Restoration, Xinjiang Institute of Ecology and Geography, Chinese Academy of Sciences, Urumqi, China; 4 Department of Agronomy, University of Agriculture, Faisalabad, Pakistan; 5 College of Biological and Food Engineering, Huanghuai University, Zhumadian, Henan, China; 6 Department of Bioresource Engineering, McGill University, Saitne-Anne-de-Bellevue, Canada; 7 University of Chinese Academy of Sciences, Beijing, China; 8 Faculty of Environmental Science and Engineering, Kunming University of Science and Technology, Kunming, China; University of Palermo, ITALY

## Abstract

*Camelina sativa* L. is an oilseed crop with wide nutritional and industrial applications. Because of favorable agronomic characteristics of *C*. *sativa* in a water-limiting environment interest in its production has increased worldwide. In this study the effect of different irrigation regimes (I_0_ = three irrigations, I_1_ = two irrigations, I_2_ = one irrigation and I_3_ = one irrigation) on physio-biochemical responses and seed yield attributes of two *C*. *sativa* genotypes was explored under semi-arid conditions. Results indicated that maximum physio-biochemical activity, seed yield and oil contents appeared in genotype 7126 with three irrigations (I_0_). In contrast water deficit stress created by withholding irrigation (I_1_, I_2_ and I_3_) at different growth stages significantly reduced the physio-biochemical activity as well as yield responses in both *C*. *sativa* genotypes. Nonetheless the highest reduction in physio-biochemical and yield attributes were observed in genotype 8046 when irrigation was skipped at vegetative and flowering stages of crop (I_3_). In genotypic comparison, *C*. *sativa* genotype 7126 performed better than 8046 under all I_1_, I_2_ and I_3_ irrigation treatments. Because 7126 exhibited better maintenance of tissue water content, leaf gas exchange traits and chlorophyll pigment production, resulting in better seed yield and oil production. Findings of this study suggest that to achieve maximum yield potential in camelina three irrigations are needed under semi-arid conditions, however application of two irrigations one at flowering and second at silique development stage can ensure an economic seed yield and oil contents. Furthermore, genotype 7126 should be adopted for cultivation under water limited arid and semi-arid regions due to its better adaptability.

## Introduction

Water resources availability to agriculture has become a critical issue in many regions of the world [1] especially in arid and semi-arid tracts and is seriously threatening crop production [2]. Industrial and domestic sector demands have taken precedence over agriculture, and have seriously threatened global food security [3]. Moreover, harsher weather and frequent drought spells have been predicted for the near future because of concerns from climate change [4]. These conditions demand introduction and assessment of drought resilient crops that can perform better under water limited environments to ensure food security.

Drought is a detrimental abiotic stress affecting productivity and quality of crop by changing plant metabolic activities and growth physiology [5]. Drought stress have negative impacts on plant water content by decreasing leaf water potential and relative water contents [6]. It alters metabolic pathways resulting in various physiological interruptions, such as transpiration loss [7], and ultimately restricts photosynthesis [8]. Furthermore, drought stress also stimulates the formation of reactive oxygen species (ROS) inside the chloroplasts, peroxisomes and mitochondria that result in cellular and metabolic dysfunction [9]. Shortage of water occurring at critical growth stages substantially decreases yield and reduces crop quality; however, different crops show different responses to drought stress at different growth stages [10]. Therefore, if a crop better adapts to water deficit conditions, then it would be a better option to strengthen the sustainable crop production systems [11].

Camelina (*Camelina sativa* L.) is an oilseed crop from *Brassicaceae* family having long history of cultivation [12]. Resurging interest in camelina lies in its attractive and unique oil and agronomic characteristics [12]. Seed oil content varies from 38–43% and protein amount lies between 27–32% [13]. Camelina oil contains α- linolenic acid (≈35%), a precursor of omega-3 fatty acids highly crucial for human well-being [14]. Camelina oil is used as biofuel and it has several important industrial applications [13]. Camelina can be cultivated successfully with supplemental irrigation in semi-arid conditions with respectable seed yield outcomes [15]. It showed better resistance to disease and drought compared to canola crop [16]. Drought tolerance potential of camelina relies upon its ability to extract water from soil that was estimated to be around 140 cm [17].

An appropriate flood irrigation management for camelina is quite necessary in water limited arid and semiarid conditions [18]. Previous studies on camelina irrigation needs under different agro climatic conditions demonstrated an increase in seed yield with higher water volumes and this information is somewhat limited [19–23]. However, no considerable attention has been paid to evaluate the impact of water deficit on physio-biochemical and yield productivity responses in camelina, when imposed at different growth stages. Since limited water supply principally harms the crop vegetative, flowering, seed setting and seed filling growth stages [24]. Additionally assessing the performance of contrasting genotypes under variable irrigation regimes will help to select suitable genotype that could perform better in water limiting conditions [22]. Since there is limited information in the literature regarding physio-biochemical expressions, seed yield, oil and protein responses against water deficit stress employed at different growth stages in camelina. Therefore the specific aims of this study were to (i) explicate the physio-biochemical responses of camelina genotypes under different irrigation regimes, (ii) recognize the crop growth stage most sensitive to limited water availability (iii) to what extent does shortage of irrigation water affect the yield and yield traits, oil and protein contents in camelina. It is assumed that outcomes of this particular study will assist in devising strategies to realize maximum yield potential of camelina in arid and semi-arid environments.

## Materials and methods

### Experimental site description

**Fig 1:**
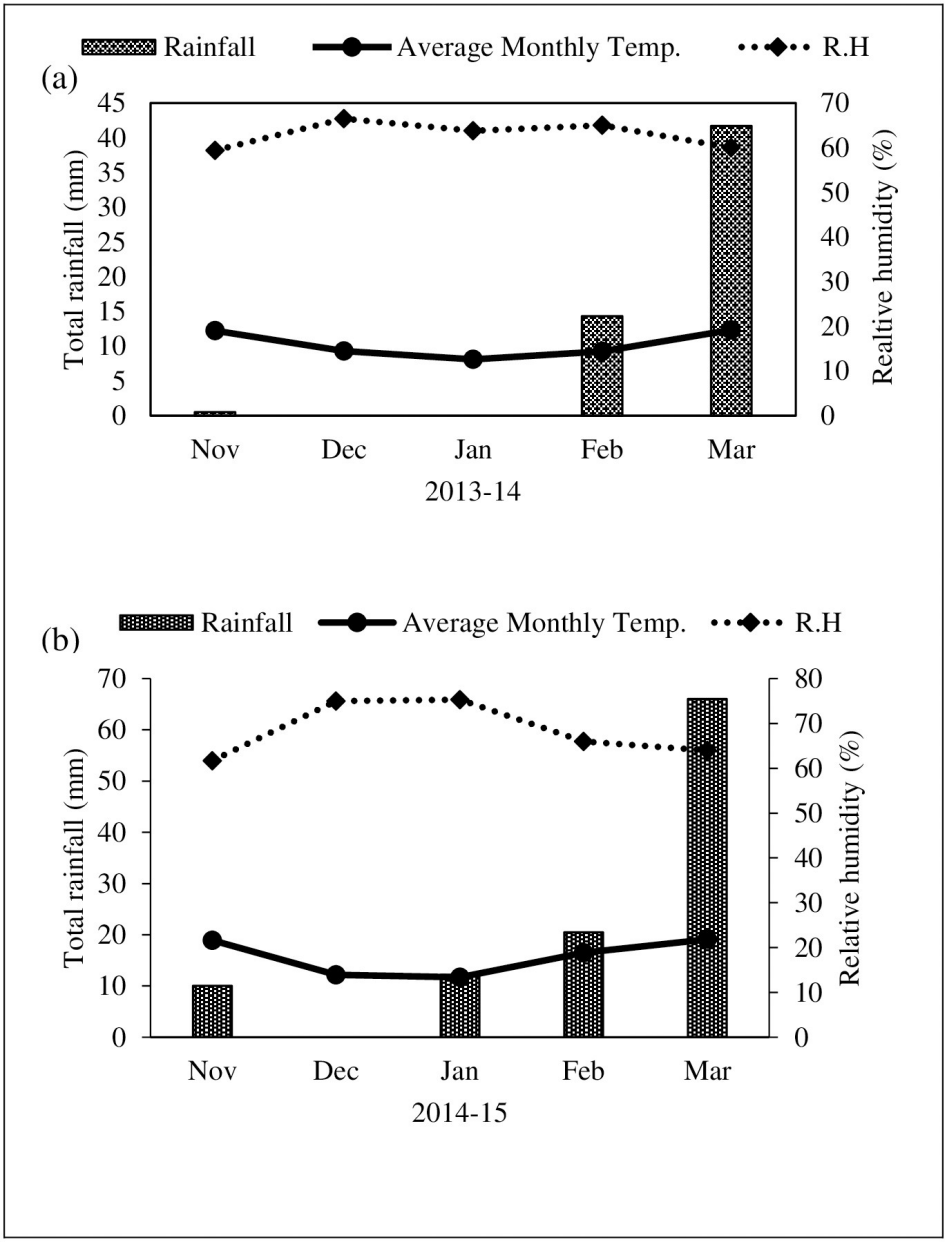
Average monthly temperature, rainfall and relative humidity during 2013–14 (a) and 2014–15 (b).

A two years field study was carried out in 2013–14 (Yr1) and 2014–15 (Yr2) (November-March) at research farm of Department of Agronomy, University of Agriculture, Faisalabad, Pakistan (31°25′ N, 73°09′ E, elevation 184.4 m). The soil at the study site was a clay loam (pH 7.5–7.8) with semi-arid climate having hot dry summer and astringent cold winter seasons (Fig 1a and 1b). The average monthly minimum temperature (5.9–13.6°C), maximum temperature (16.6–26.3°C) and relative humidity (59–75%) (Fig 1a and 1b) did not vary significantly during both growing seasons respectively, however rainfall was higher (108 mm) in 2014–15 compared to 2013–14 (56.5 mm). A complete physico-chemical analysis of soil was performed and presented in Table 1. Soil analysis were performed in the soil analysis lab of Institute of Soil and Environmental Sciences, University of Agriculture, Faisalabad. Standard procedures described in Hand book No. 60 [25] were followed, while available P and soil texture were determined by the methods illustrated by Watanabe and Olsen [26] and Moodie et al. [27]. Two camelina genotypes (7126 and 8046) were evaluated in this study, seeds were acquired from the Office of Research, Innovation and Commercialization (ORIC), University of Agriculture, Faisalabad, Pakistan. The irrigation treatments included normal irrigation (Control = I_0_) with three irrigations (one irrigation at vegetative stage (30 days after seeding, DAS), second irrigation at flowering (55 DAS) and third irrigation at silique (pod) development stage (75 DAS), (I_1_) with two irrigations, (one irrigation at flowering (55 DAS) and second at silique development stage (75 DAS), (I_2_) with only one irrigation at flowering stage (55 DAS) and (I_3_) with one irrigation at silique development stage (75 DAS), respectively. The experimental layout was randomized complete block design and replicated thrice. The experimental plot size was 5 m × 6 m, which were prepared for seeding with an initial pre-sowing irrigation to bring the soil to field capacity and soil moisture content was 22%. Gravimetric soil water samples were taken to estimate soil moisture content. The amount of irrigation water applied was measured by a cutthroat flume. A 90 cm cutthroat flume having 20 cm wide throat was installed in the center of the water channel releasing 76 mm per irrigation event. The discharge of water was calculated from the free-flow calibration table. The time of irrigation was calculated from the following formula [28].

$$t=(\frac{A\times d}{Q})$$

Where t is the time (seconds) of irrigation for a given area, A is the area to be irrigated in m^2^, and d is the depth (mm) of water applied. Q is the discharge from flume in m^3^ s^−1^. Table 2 represents the data about quantity of irrigation water applied during both growing seasons.

**Table 1:** Physiochemical characteristics of the soil during 2013–14 and 2014–15.

Soil Characteristics	2013–14	2014–15
Soil texture	Clay loam	Clay loam
Saturation percentage (%)	36.6	35.8
Soil pH	7.5–7.8	7.6–7.8
Organic matter (%)	0.4–0.6	0.4–0.5
EC_e_ (dSm^−1^)	0.76–0.92	0.73–0.91
HCO_3_ (meq L^−1^)	3.6–4.2	3.5–4
Ca+Mg (meq L^−1^)	4.1–5.68	3.5–5.6
CO_3_ (meq L^−1^)	Nil	Nil
NO_3_-N (mg kg^−1^)	11.1–13.8	12–14.2
Available P (mg kg^−1^)	8.5–10.8	8.3–9.9
Available K (mg kg^−1^)	112–200	110–195

**Table 2:** Amount of irrigation water (mm) applied under different irrigation regimes (I_0_, I_1_, I_2_ and I_3_) in camelina during 2013–14 and 2014–15.

**2013–14**					
**Irrigation treatments**	**Vegetative stage (30 DAS)**	**Flowering stage (55DAS)**	**Silique development stage (75 DAS)**	**Rainfall (mm)**	**Total (mm) (Irrigation +Rain fall)**
I_0_	76	76	76	56.5	284.5
I_1_	×	76	76	56.5	208.5
I_2_	×	76	×	56.5	132.5
I_3_	×	×	76	56.5	132.5
**2014–15**					
I_0_	76	76	76	108	336
I_1_	×	76	76	108	260
I_2_	×	76	×	108	184
I_3_	×	×	76	108	184

DAS represents days after seeding and × indicates no irrigation application.

Prior to seeding two tilling operations were performed with a tractor mounted cultivator followed by a planking operation. Both cultivars were sown on November 14, 2013 and November 18, 2014 using a hand-operated drill with a row spacing of 30 cm. After plant emergence (15 DAS) thinning was done to establish 10 cm spacing between plants. Nitrogen (N) and phosphorus (P_2_O_5_) fertilizers were manually applied at 100 kg ha^−1^ and 30 kg ha^−1^ using urea and di-ammonium phosphate as sources, respectively. Two irrigation treatments (I_0_ and I_1_) received all the P and half the N at the time of sowing and the other half of N with the first irrigation. The other two irrigation treatments (I_2_ and I_3_) received all the N and P at the time of sowing. Weed control was done twice using a hand hoe. The plots were irrigated through flood irrigation method. Data regarding leaf gas exchange traits, leaf water relations, pigment estimation was recorded 50 days after sowing, while yield and yield related parameters were noted after crop being harvested on 15^th^ and 18^th^ March each year respectively.

### Leaf water relations

Five fully expanded leaves from each treatment were selected to record leaf water potential (*Ψw*) using a hand-held pressure chamber (ARIMAD-A, ELE-International by MRC) technique [29]. The same leaves were packed in re-sealable plastic bags and kept in a biomedical freezer (Sanyo Freezer MDF-U730) for one week at -20°C. The frozen leaves were then thawed and crushed with a glass rod to extract the sap and take osmotic potential readings (*Ψs*) with an osmometer (Wescor, 5520). Calculation of leaf turgor pressure (*Ψp*) was done by using the following formula [30].

(Ψp)=(Ψw)−(Ψs)

Where

Ψp = Leaf turgor potential

Ψw = Leaf water potential

Ψs = Leaf osmotic potential

### Leaf gas exchange rates

Leaf gas exchange rates net photosynthetic rate (*P*_*N*_), transpiration (*E*) and stomatal conductance (*G*_*s*_) were recorded from the fully expanded top three leaves of five plants from each plot with a portable infrared gas analyzer (CI-340, CID, Bio Science, Inc.). All observations were noted between 9:00–11:00 a.m. with following regulations: molar flow of air per unit of leaf area 401mmol m^−2^ s^−1^, atmospheric pressure 98.7kPa, PAR up to 1690 μmol m^−2^ s^−1^, water vapor pressure ranged from 6.2 to 8.7 mbar, leaf temperature (28°C), ambient temperature (23 to 27°C) and ambient CO_2_ amount was 352 mol mol^−1^.

### Leaf chlorophyll contents

Leaf chlorophyll (Chl *a*, Chl *b* and Chl *t*) concentration was measured on 1 gram fresh leaf samples taken from five randomly selected plants from each plot. After chopping into small pieces, each leaf sample was extracted with 20 ml acetone (80%) and kept overnight at 4°C in a refrigerator [31]. These samples were then centrifuged at 500 rpm for 5 minutes and properly filtered The supernatant absorbance was estimated at wavelengths of 645 and 663nm on spectrophotometer (Hitachi U2000, Japan) by using the following formula;

Chla=(12.7OD663−2.69OD645)×V/1000W

Chlb=(22.9OD645−4.68OD663)×V/1000W

Where V represents the volume of sample extract and W is the weight of the sample.

### Yield and yield related traits

Camelina was hand harvested when 90% of the pods had turned brown. Above ground biomass from 1 m^2^ was hand harvested in each plot, dried, weighed and threshed for total biomass and seed yield. Plant height (cm), number of branches per plant and number of pods per plant were recorded separately from ten randomly selected plants of each plot. Thousand-seed weight (g) was determined and harvest index was calculated with following formula [32]

$$\text{Harvest}\;\text{index}\;(\text{HI})\;=\frac{\text{Seed}\;\text{yield}}{\text{Biological}\;\text{yield}}\times 100$$

Where biological yield means total dry biomass (grain + straw) produced by the crop.

### Seed oil and protein contents

Oil and protein contents from 5.0 g of whole C. *sativa* seeds taken from each replication were derived (on a dry matter basis) using near-infrared reflectance spectroscopy (NIRS) using a Model 6500 NIR spectrophotometer [33]. NIR calibration was performed by following the standard procedure described by Velasco et al. [34]. Both oil and protein yields (kg ha^−1^) of camelina were calculated by multiplying the percentage of seed oil and protein content with seed yield [35].

### Statistical analyses

Data were statistically analyzed with Fisher’s analysis of variance (ANOVA) technique. Three-way ANOVA was used to determine the effects of irrigation, genotype and year and their interactions on plant properties using Statistix-9.1 software. Least significant difference (LSD) test with 5% probability level was used to make treatment mean comparisons [36]. Microsoft excel program was used to develop the figures and calculation of standard error and correlation coefficients.

## Results

### Leaf water relations

**Fig 2:**
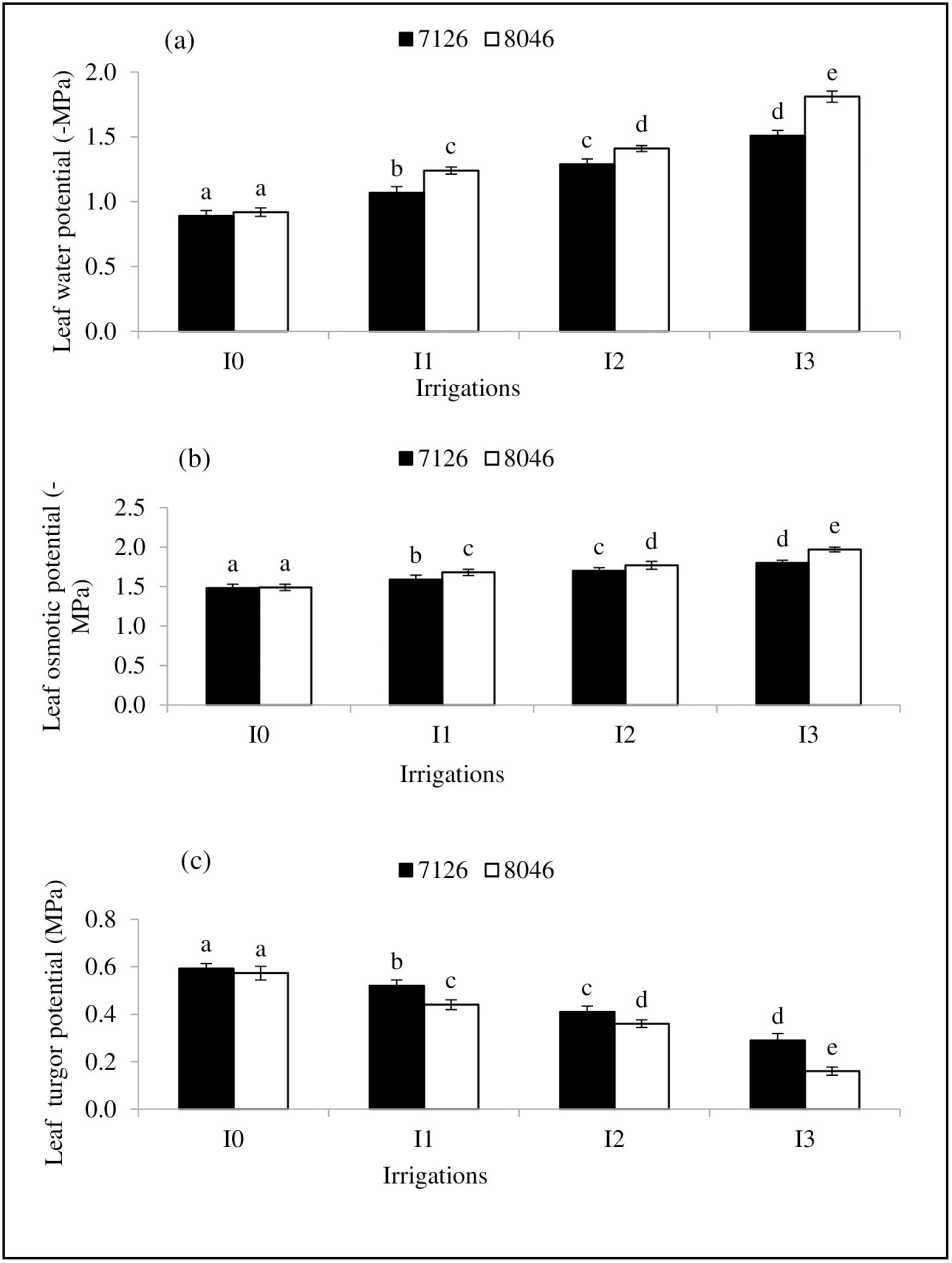
Effect of different irrigation regimes (I_0_, I_1_, I_2_ and I_3_) on (a) leaf water potential (*Ψw*), (b) leaf osmotic potential (*Ψs*) and (c) leaf turgor potential (*Ψp*) of *Camelina* genotypes (7126 and 8046). Bars (Mean ± SE) carrying different letters represent significant variation at *P≤*0.05.

Leaf water potential (Ψw), solute potential (Ψs) and pressure potential (Ψp) were significantly (*P≤*0.05) affected by irrigation regimes (I), genotypes (G) and their interaction (I×G), while year effect was non-significant (Table 3). Maximum values of Ψw (-0.89 MPa), Ψs (-1.48 MPa) and Ψp (0.59 MPa) were observed under normal irrigation (I_0_) in genotype 7126 and it was statistically at par with genotype 8046 (Fig 2a–2c). Minimum values of Ψw (-1.81MPa), Ψs (-1.97 MPa) and Ψp (0.16 MPa) were recorded with irrigation treatment (I_3_) in genotype 8046 compared to normal irrigation (Fig 2a–2c). Imposition of water deficit stress by skipping irrigation at critical growth stages (I_1_, I_2_ and I_3_) of crop significantly (*P≤*0.05) reduced the Ψw, Ψs and Ψp values in both genotypes. However genotype 7126 showed less reduction in Ψw, Ψs and Ψp values compared to genotype 8046 under water deficit conditions (I_1_, I_2_ and I_3_) (Fig 2a–2c). Hence genotype7126 showed better adaptability under restricted water supply (I_1,_ I_2_ and I_3_) by sustaining better leaf water contents compared to 8046.

**Table 3:** Analysis of variance (ANOVA) F-test significance of genotypes, irrigations and years regarding yield and yield related traits of camelina during 2013–14 and 2014–15.

Factors	DF	Ψw	Ψs	Ψp	Chl-*a*	Chl-*b*	Chl-*t*	*P*_*N*_	*E*	*G*_*s*_
Genotype (G)	1	**	**	**	**	**	**	**	**	**
Irrigation (Irr)	3	**	**	**	**	**	**	**	**	**
Year (Y)	1	ns	ns	ns	ns	*	*	*	ns	ns
G×Irr	3	*	*	*	*	*	*	*	*	*
G×Year	1	ns	ns	ns	ns	ns	ns	ns	ns	ns
Irr×Y	3	ns	ns	ns	ns	ns	ns	ns	ns	ns
G×Irr×Year	3	ns	ns	ns	ns	ns	ns	ns	ns	ns
CV (%)		3	2.14	6.87	2.37	4.78	2.58	5.22	7.75	6.18

*, ** significant at p ≤ 0.05 and p ≤ 0.01 respectively;

ns = non-significant

### Chlorophyll contents

**Fig 3:**
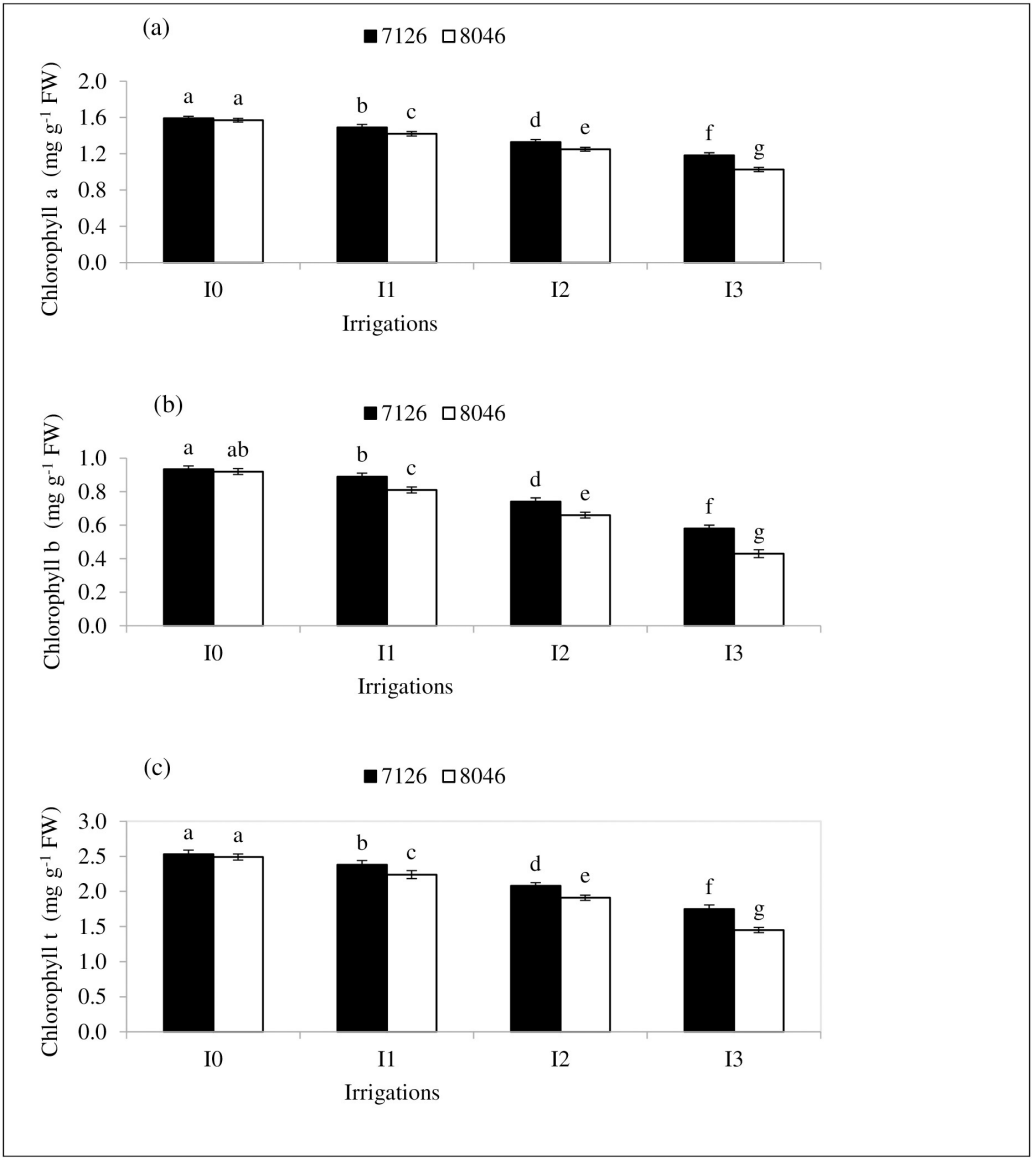
Effect of different irrigation regimes (I_0_, I_1_, I_2_ and I_3_) on (a) chlorophyll a, (b) chlorophyll b and (c) total chlorophyll contents of *Camelina* genotypes (7126 and 8046). Bars (Mean ± SE) carrying different letters represent significant variation at *P≤*0.05.

The results indicated that leaf chlorophyll contents (Chl *a*, Chl *b* and Chl *t*) were significantly affected by irrigation regimes (I), genotypes (G) and their interaction (I×G) (Table 3). Camelina genotypes 7126 revealed the highest Chl *a* (1.59 mg g^−1^ FW), Chl *b* (0.93 mg g^−1^ FW) and Chl *t* (2.52 mg g^−1^ FW) contents under normal irrigation (I_0_) and remain statistically at par with genotype 8046 (Fig 3a–3c). The lowest values of Chl *a* (1.02 mg g^−1^ FW), Chl *b* (0.43 mg g^−1^ FW) and Chl *t* (1.45 mg g^−1^ FW) were recorded in genotype 8046 under I_3_ irrigation treatment compared to normal irrigation (I_0_) (Fig 3a–3c). Withholding irrigation at critical growth stages (I_1,_ I_2_ and I_3_) induced significant (*P≤*0.05) reduction in the concentration of chlorophyll contents (Chl *a*, Chl *b* and Chl *t*) of both genotypes. But the magnitude of this reduction was less in genotype 7126 compared to 8046. These results imply that chlorophyll contents (Chl *a*, Chl *b* and Chl *t*) were less affected in genotype 7126 compared to 8046 under water deficit stress (I_1,_ I_2_ and I_3_).

### Leaf gas exchange traits

**Fig 4:**
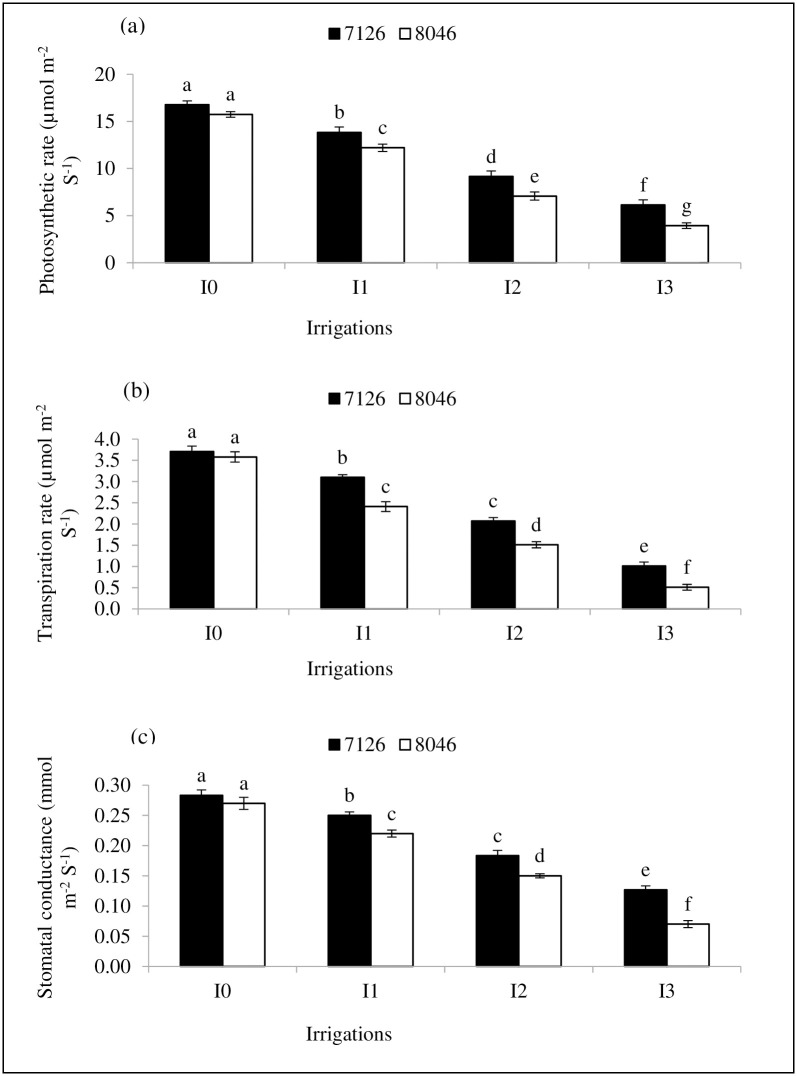
Effect of different irrigation regimes (I_0_, I_1_, I_2_ and I_3_) on (a) photosynthetic rate (*P*_*N*_), (b) transpiration rate (*E*) and (c) stomatal conductance (*G*_*s*_) of *Camelina* genotypes (7126 and 8046). Bars (Mean ± SE) carrying different letters represent significant varitaion at *P≤*0.05.

Camelina genotypes (7126 and 8046) subjected to water deficit stress imposed by withholding irrigation at critical growth stages (I_1,_ I_2_ and I_3_) demonstrated significant (*P≤*0.05) decrease in leaf gas exchange traits *P*_*N*_, *E* and *G*_*s*_ (Fig 4a–4c). However, maximum *P*_*N*_ (16.78 μmol m^−2^s^−1^), *E* (3.71 μmol m^−2^s^−1^) and *G*_*s*_ (0.28 mmol m^−2^s^−1^) values were noted in normal irrigation (I_0_) with genotype 7126 and it did not differ significantly (*P≤*0.05) from genotype 8046 (Fig 4a–4c). The treatment combination I_3_ and genotype 8046 revealed the lowest values of *P*_*N*_ (3.94 μmol m^−2^s^−1^), *E* (0.51 μmol m^−2^s^−1^) and *G*_*s*_ (0.07 mmol m^−2^s^−1^) as compared to normal irrigation (Fig 4a–4c). Overall genotype 7126 maintained better values of leaf gas exchange attributes compared to genotype 8046 in response of water deficit stress (I_1,_ I_2_ and I_3_) imposed by skipping irrigation.

### Yield and yield related traits

Maximum plant height (117.6 cm), number of branches per plant (18), number of pods per plant (561), 100-seed weight (1.7g), biological yield (8.3 t ha^−1^), seed yield (1.9 t ha^−1^) and harvest index (23.3%) were recorded in genotype 7126 under normal irrigation (I_0_), while these responses were statistically similar with genotype 8046 (Table 5). Minimum value of plant height (40.3 cm), number of branches per plant (5), number of pods per plant (225), 100-seed weight (0.92 g), biological yield (4.5 t ha^−1^), seed yield (0.54 t ha^−1^) and harvest index (12%) were recorded in genotype 8046 under I_0_ irrigation treatment as compared to normal irrigation (I_0_) (Table 5). Both genotypes 7126 and 8046 manifested significant (*P≤*0.05) decrease (Table 5) in in terms yield traits under I_1,_ I_2_ and I_3_ irrigation regimes but genotype 7126 appeared to be less affected by water deficit stress as compared to genotype 8046.

### Seed oil and protein contents

Different irrigation regimes (I) and camelina genotypes (G) represented significant (*P≤*0.05) individual and interactive (I×G) effects regarding seed oil and protein contents (Table 4). The maximum seed oil content (40%) was found in camelina genotype 7126 under normal irrigation treatment (I_0_) and it was statistically at par with genotype 8046 (Table 5). Decreasing availability of soil water due to withholding irrigation at critical growth stages (I_1,_ I_2_ and I_3_) caused significant decrease in seed oil content and the lowest seed oil content was noted in genotype 8046 under I_3_ irrigation regime as compared to normal irrigation (Table 5). Nevertheless genotype 7126 demonstrated better seed oil content with deficit water supply (I_1,_ I_2_ and I_3_) compared to 8046 genotype.

**Table 4:** Analysis of variance (ANOVA) F-test significance of genotypes, irrigations and years regarding leaf water relations, chlorophyll pigments and leaf gas exchange traits of camelina during 2013–14 and 2014–15.

Factors	DF	Plant height	Branches/plant	Pods/plant	1000-Seed weight	Biological yield	Seed yield	Harvest index	Seed oil	Seed protein
Genotype (G)	1	**	**	**	**	**	**	**	**	**
Irrigation (Irr)	3	**	**	**	**	**	**	**	**	**
Year (Y)	1	**	ns	ns	ns	ns	ns	ns	ns	ns
G×Irr	3	*	*	*	*	*	*	*	*	*
G×Y	1	ns	ns	ns	ns	ns	ns	ns	ns	ns
Irr×Year	3	ns	ns	ns	ns	ns	ns	ns	ns	ns
G×Irr×Y	3	ns	ns	ns	ns	ns	ns	ns	ns	ns
CV (%)		6.24	10	4.26	4.56	2.60	5.08	2.78	6.80	3.37

*, ** significant at p ≤ 0.05 and p ≤ 0.01 respectively;

ns = non-significant

**Table 5:** Mean comparison (± SE) of two-way interactions between irrigation regimes (I) and camelina genotypes (G) on yield and yield related traits (three replications) of camelina.

Irrigation regimes (I)	Camelina genotypes (G)	Plant height (cm)	Branches/plant	Pods/plant	1000-Seed weight (g)	Biological yield (t h^−1^)	Seed yield (t h^−1^)	Harvest Index (%)	Seed Oil Content (%)	Seed Protein (%)
I_0_	7126	116.5±2.69a	18±0.85a	561±11.40a	1.7±0.05a	8.3±0.10a	1.9±0.04a	23.3±0.11a	39.4±0.96a	19.4±0.20d
	8046	113.6±2.99a	17±0.87a	541±8.59a	1.7±0.04a	8.1±0.11a	1.8±0.05a	22.8±0.23a	37.5±0.95b	19.2±0.81d
I_1_	7126	102.9±2.78b	15±0.68b	476±11.90b	1.5±0.03b	7.7±0.07b	1.6±0.04b	21.3±0.28b	36.2±1.37b	24.5±0.53c
	8046	93.6±3.79c	13±0.57c	431±6.34c	1.4±0.05c	6.8±0.09c	1.3±0.06c	19.3±0.56c	33.1±1.11c	20.8±0.62d
I_2_	7126	82.0±2.91d	12±1.08c	429±12.30c	1.4±0.04c	6.7±0.10c	1.2±0.05c	17.9±0.37d	30.3±0.97d	28.1±0.38b
	8046	65.1±2.80e	9±0.67d	335±6.92d	1.1±0.05d	5.8±0.08d	0.78±0.03d	14.6±0.19e	23.8±1.22e	25.2±0.53c
I_3_	7126	60.5±4.92 e	8±0.68d	308±6.41d	1.2±0.07d	4.8±0.09e	0.72±0.05d	14.3±0.35e	25.0±1.15e	31.6±0.39a
	8046	40.3±1.96f	5±0.67e	225±8.02e	0.92±0.04e	4.5±0.11f	0.54±0.06e	12.0±0.20f	17.2±0.88f	28.3±0.37b
LSD		5.69	1.32	21	0.07	0.20	0.08	0.61	1.73	0.98

Values carrying different letters in each column represent statistically significant difference among treatments at p≤0.05.

In case of seed protein content, an increase was observed with decreasing availability of water. Maximum seed protein content (31.6%)was found in genotype 7126 under I_3_ irrigation regime while minimum value (19.2%) of seed protein content was recoded under normal irrigation (I_0_) with genotype 8046 which was statistically similar to 7126 (19.4%) (Table 5). Although imposition of water deficit stress (I_1,_ I_2_ and I_3_) induced significant rise in seed protein content of both genotypes, but this rise was significantly higher in genotype 7126 compared to genotype 8046 under I_1,_ I_2_ and I_3_ irrigation regimes.

### Correlation among physio-biochemical attributes, agronomic traits, seed oil and protein contents

Correlation data presented in Table 6, indicated strong positive correlation of leaf water relations (Ψw, Ψs and Ψp), chlorophyll contents (Chl *a*, Chl *b* and Chl *t*), leaf gas exchange rates (*P*_*N*_, *E* and *g*_*s*_) with seed yield and yield related traits and seed oil contents. However, seed protein contents exhibited a strong negative correlation with all the above mentioned traits.

**Table 6:** Correlation coefficients of leaf water relations, chlorophyll contents, leaf gas exchange traits, seed yield components, seed oil and protein contents of camelina under different irrigation regimes.

**Ψw**																	
**Ψs**	0.98***																
**Ψp**	0.97***	0.94***															
**Chl *a***	0.98***	0.96***	0.94***														
**Chl *b***	0.97***	0.97***	0.97***	0.99***													
**Chl *t***	0.96***	0.98***	0.95***	0.96***	0.95***												
***P***_***N***_	0.99***	0.95***	0.98***	0.98***	0.98***	0.93***											
***E***	0.96***	0.96***	0.96***	0.95***	0.93***	0.95***	0.95***										
**g**_**s**_	0.94***	0.95***	0.92***	0.97***	0.96***	0.98***	0.97***	0.97									
**PH**	0.98***	0.94***	0.97***	0.92***	0.94***	0.94***	0.98***	0.94	0.91***								
**BR**	0.96***	0.97***	0.93***	0.93***	0.97***	0.96***	0.96***	0.95	0.94***	0.96***							
**Pods**	0.95***	0.93***	0.97***	0.97***	0.91***	0.93***	0.98***	0.93	0.94***	0.98***	0.9***						
**SW**	0.97***	0.96***	0.98***	0.95***	0.97***	0.94***	0.94***	0.91	0.96***	0.95***	0.91***	0.93***					
**BY**	0.95***	0.95***	0.94***	0.96***	0.93***	0.96***	0.98***	0.94	0.92***	0.92***	0.93***	0.95***	0.97***				
**SY**	0.93***	0.91***	0.93***	0.94***	0.98***	0.97***	0.95***	0.97	0.97***	0.93***	0.94***	0.98***	0.95***	0.96***			
**HI**	0.97***	0.96***	0.95***	0.97***	0.96***	0.97***	0.97***	0.96	0.97***	0.92***	0.96***	0.96***	0.91***	0.93***	0.93***		
**Oil**	0.95	0.98***	0.97***	0.92***	0.93***	0.95***	0.95***	0.97	0.94***	0.89***	0.97***	0.95***	0.93***	0.96***	0.95***	0.94***	
**Protein**	-0.97***	-0.98***	-0.96***	-0.95***	-0.92***	-0.93***	-0.96***	-0.94	-0.95***	-0.91***	-0.94***	-0.96***	-0.94***	-0.92***	-0.96***	-0.97***	-0.94***

*** Significant at p<0.001;

PH = Plant height, BR = Branches, SW = Seed weight, BY = Biological yield, SY = Seed yield, HI = Harvest index,

## Discussion

### Leaf water relations

Results of this study demonstrated that imposing soil water deficit stress by withholding irrigation at different growth stages considerably reduced the leaf water (Ψw), solute (Ψs) and pressure (Ψp) potentials in both camelina genotypes with the highest reduction occurring in genotype 8046 under I_3_. These results are in line with those of Norouzi et al. [37] and Shekari et al. [38], who reported a notable reduction in leaf water status of rapeseed cultivars in response of limited irrigation at early vegetative and flowering growth stages. However, this decrease in water potential traits could be attributed to limited availability of soil moisture [39, 40]. Decline in Ψw could have been a major defense strategy of plants to tolerate drought conditions by retaining water and osmolytes to an acceptable level [41]. Such decrease in Ψs creates a gradient of water movement into the plant cells which could help to sustain cell turgor [42]. Differential response of camelina genotypes regarding leaf water relations would be due to their contrasting ability of water uptake from soil under water deficit conditions because drought resilience feature of camelina is correlated with its ability to extract water from deeper soil layers [17].

### Chlorophyll contents

Water limited conditions significantly reduced the concentration of chlorophyll pigments (Chl *a*, Chl *b* and Chl *t*) in both genotypes compared to the normal irrigation (I_0_). These results are in line with the reports of [29, 43] who reported same reduction pattern in chlorophyll contents due to drought stress. This reduction in leaf chlorophyll contents might be the result of drought induced production of reactive oxygen species (ROS) [44]. Reactive oxygen species damages the chloroplast, cause changes in the ultrastructure of plastids, thylakoid membranes and also induce lipid peroxidation [45, 46]. These alterations might have disrupted the normal functioning of enzymes responsible for chlorophyll biosynthesis [39, 47]. Consequently chlorophyll pigment production has been reduced under drought stress. Higher reduction of chlorophyll contents in genotype 8046 could be attributed to its lower resistance against drought induced oxidative damage [47, 48]. Whereas, the maintenance of higher chlorophyll contents in genotype 7126, might be a manifestation of relatively more resistant and stable ultra-structure of its photosynthetic machinery under drought stress [49].

### Leaf gas exchange rates

Drier soil conditions due to decreased irrigation significantly reduced the performance of leaf gas exchange attributes (*P*_*N*_, *E* and *G*_*s*_) in both camelina genotypes compared to normal irrigation (I_0_). Results of this study are in agreement with [50] who observed a similar decrease in leaf gas exchange traits of camelina and cotton [51] under water limited conditions. This decrease in the performance of photosynthetic gas exchange traits appears to be the consequence of chlorophyll destruction and stomatal limitations in response to water shortages [29, 52]. Reduction in *P*_*N*_ might have occurred due to chlorophyll disintegration, stomatal closure and metabolic impairments that could have restricted the carbon fixation under deficient supply of water [29, 50, 53]. Because stomatal closure reduces photosynthetic rate by decreasing internal CO_2_ concentration, Rubisco activity and ATP synthesis under water deficit conditions [54]. Moreover, decrease in *E* and *G*_*s*_ might have also occurred due to stomatal closure as its a defensive response of the plant under drought stress to conserve water by minimizing water loss through the stomata [55]. Relatively higher values of *P*_*N*_, *E* and *G*_*s*_ in genotype 7126 compared to 8046 could be the result of more efficient and better stomatal control of genotype 7126 to manage water loss by transpiration under limited soil moisture conditions [56].

### Yield and yield related traits

It has also been observed that decreasing soil water availability under I_1_, I_2_ and I_3_ irrigation treatments caused significant reduction in yield and development of yield contributing traits in camelina genotypes during both growing seasons. These results are consistent with the findings of [20–22]. The highest decrease in yield and yield contributing traits of both genotypes was observed when irrigation was skipped at vegetative and flowering stages (I_3_). However, this reduction could have been the result of declined tissue water content, reduced chlorophyll pigment production, and substantially decreased photosynthetic rate resulting in poor development of yield contributing attributes under soil moisture deficit conditions [29, 57]. Because, it is believed that less photosynthetic activity in dicots usually transfers fewer assimilates to the developing reproductive structures which leads to prominent yield loss [58]. Yet, in the present elucidation, limited supply of assimilates to yielding fractions would have led to seed shrinkage and loss of seed weight resulting in substantial yield reduction [59, 60]. Besides, reduction in yield could also be associated with lesser number of pods, fewer flowers, pod abortion and shortening of the seed filling period with no irrigation at flowering stage [61]. Likewise, short supply of water also caused notable decrease in biological yield, seed yield and harvest index of camelina genotypes. These results are in agreement with [29] who also reported similar decreasing in yielding response of camelina under soil moisture deficient conditions. Overall, the yield and yielding traits performance of camelina genotype 7126 was found comparatively better than genotype 8046 at all irrigation treatments, and this could be attributed to better physiological and biochemical adaptations of 7126 to declining soil moisture regimes.

### Seed oil and protein contents

Seed oil content decreased, while seed protein concentration increased in both camelina genotypes under water deficit conditions (I_1_, I_2_ and I_3_ irrigation regimes). In the line of these findings, earlier studies have also reported similar contrasting relationship between the said parameters [62, 63]. Possibly it may have happened due to the reduced supply of carbohydrates (photosynthates) to developing seeds, needed for triacylglycerol biosynthesis, resulting in the lower accumulation of seed oil [64]. Besides that, it is further argued that higher conversion and deposition of available assimilates into proteins during seed development, could have declined the conversion of metabolites (assimilates) into oil under limited water conditions, leading to relatively reduced oil deposition in seeds [65, 66]. Outcomes of the current study are consistent with the findings of [29], showing a notable decline in oil content and a concurrent rise in protein content in camelina genotypes under drought stress. On the other hand, differential behavior of camelina genotypes regarding seed oil and protein contents under water shortage conditions might be the result of differences in their genetic makeup [67]. These results suggested that genotype 7126 better utilized the available carbon assimilate resources in seed oil and protein biosynthesis under water deficit conditions compared to 8046.

Oil and protein yields (kg ha^−1^) of camelina (S1 and S2 Figs) also decreased under water deficit conditions (I_1_, I_2_ and I_3_) compared to normal irrigation (I_0_). These results are in accordance with the previous findings, revealing a significant decrease in oil and protein yields of camelina [20–22] and canola [30] under limited supply of water. The possible reason of this reduction in oil and protein yields would be the decrease in seed yield because oil and protein yields are the output of seed oil and protein contents multiplied with seed yield [30].

## Conclusion

Findings of this study suggested that water deficit stress created by withholding irrigation (I_1_, I_2_ and I_3_), at different growth stages considerably reduced the physio-biochemical responses, seed yield and oil contents in both camelina genotypes (7126 and 8046) compared to normal irrigation (I_0_). Regarding irrigation treatments (I_1_, I_2_ and I_3_), water deficit at vegetative and flowering stages (I_3_) caused the maximum reduction in physio-biochemical responses, seed yield and oil contents of genotype 8046. However, genotype 7126 relatively performed better under (I_1_, I_2_ and I_3_) irrigation regimes regarding above said parameters compared to 8046. It is suggested that to achieve maximum yield and oil potential camelina should be grown with three irrigations under arid and semi-arid regions, however under water limited conditions economic seed yield and oil contents could be obtained with two irrigations (I_1_). Moreover, genotype 7126 would be a better choice for cultivation in water limited arid and semi- arid regions due to it better endurance.

## Supporting information

S1 Fig Effect of different irrigation regimes (I_0_, I_1_, I_2_ and I_3_) on oil yield (kg ha^−1^) of *Camelina* genotypes (7126 and 8046). Bars carrying different letters represent significant variation at *P≤*0.05.
(DOCX)

S2 Fig Effect of different irrigation regimes (I_0_, I_1_, I_2_ and I_3_) on protein yield (kg ha^−1^) of *Camelina* genotypes (7126 and 8046). Bars carrying different letters represent significant variation at *P≤*0.05.
(DOCX)
